# ECM Mimetic Electrospun Porous Poly (L-lactic acid) (PLLA) Scaffolds as Potential Substrates for Cardiac Tissue Engineering

**DOI:** 10.3390/polym12020451

**Published:** 2020-02-14

**Authors:** Priyadharshni Muniyandi, Vivekanandan Palaninathan, Srivani Veeranarayanan, Tomofumi Ukai, Toru Maekawa, Tatsuro Hanajiri, Mohamed Sheikh Mohamed

**Affiliations:** 1 Graduate School of Interdisciplinary New Science, Toyo University, Kawagoe, Saitama 350-8585, Japan; priyadharshni8612@gmail.com (P.M.); tmfmukai@toyo.jp (T.U.); maekawa@toyo.jp (T.M.); hanajiri@toyo.jp (T.H.); 2 Bio-Nano Electronics Research Centre, Toyo University, Kawagoe, Saitama 350-8585, Japan; vivekanandan@toyo.jp (V.P.); srivani528@toyo.jp (S.V.)

**Keywords:** cardiac tissue engineering, electrospinning, porous polymeric fibers, cell scaffold, extracellular matrix

## Abstract

Cardiac tissue engineering (CTE) aims to generate potential scaffolds to mimic extracellular matrix (ECM) for recreating the injured myocardium. Highly porous scaffolds with properties that aid cell adhesion, migration and proliferation are critical in CTE. In this study, electrospun porous poly (l-lactic acid) (PLLA) porous scaffolds were fabricated and modified with different ECM derived proteins such as collagen, gelatin, fibronectin and poly-L-lysine. Subsequently, adult human cardiac fibroblasts (AHCF) were cultured on the protein modified and unmodified fibers to study the cell behavior and guidance. Further, the cytotoxicity and reactive oxygen species (ROS) assessments of the respective fibers were performed to determine their biocompatibility. Excellent cell adhesion and proliferation of the cardiac fibroblasts was observed on the PLLA porous fibers regardless of the surface modifications. The metabolic rate of cells was on par with the conventional cell culture ware while the proliferation rate surpassed the latter by nearly two-folds. Proteome profiling revealed that apart from being an anchorage platform for cells, the surface topography has modulated significant expression of the cellular proteome with many crucial proteins responsible for cardiac fibroblast growth and proliferation.

## 1. Introduction

Myocardial infarction—or ‘heart attack’—is one of the leading causes of death worldwide, which ensues upon the blockage of coronary artery that cuts oxygen supply to the heart muscles, which adversely affects cell orientation and often leads to the injury or death of myocytes [1]. Tissue engineering offers a promising avenue to mimic the extracellular matrix (ECM), where the potential use of biomaterial-based scaffolds can improve cell retention, survival and differentiation while also promoting adhesion, proliferation and migration with an appropriate microenvironment [2,3]. Numerous kinds of scaffolds from both natural and synthetic biomaterials have been exploited as a potential substrate for cardiac tissue engineering. Though the natural biomaterials have superior biocompatibility, their inconsistency in fabrication and lack of key characteristics such as degradation rate, mechanical, physical properties that are ideal for a scaffold have limited their use [4]. Alternatively, a synthetic biomaterial-based scaffold is expedient due to its excellent mechanical property, pure chemical composition and degradation rate, however these synthetic scaffolds lack biological recognition [5,6]. Thus, requiring an ideal scaffold to closely mimic both the structural and biochemical microenvironment of ECM.

To overcome these challenges in the field of cardiac tissue engineering, fabrication of a scaffold as a 3D support to closely mimic cardiac ECM for better cell-cell communication is essential. Electrospinning is a robust, simple and economical technique for fabricating fibers with high surface area to volume ratio, facilitating cell infiltration, which is ideal for cardiac tissue engineering [7]. The electrospun fibers have enormous potential in regenerative medicine due to their capability to mimic the naïve microenvironment [8,9]. These fibers have a substantial influence on the cell orientation, proliferation, migration and function of cells by providing more interconnecting spaces [10]. However, material consideration and chemical composition of the scaffolds are crucial factors, which influence cell adhesion, orientation and protein adhesion. In the case of cardiac tissue engineering and regeneration, an ideal scaffold should possess superior mechanical and elastic properties that are crucial for the cell growth. Among several synthetic polymers, poly (L-lactic acid) (PLLA) has been widely used to make electrospun fibers, due to its desirable properties such as cytocompatibility, biodegradability and large specific surface area with controllable pore size. The chirality of lactic acid (L and D) greatly influences the thermal, mechanical and degradation properties [11]. However, pure PLLA nanofibers due to their surface properties have been unsuitable for cell adhesion and proliferation [12,13,14,15]. In order to enhance the cell attachment to the synthetic matrix, various modifications have been developed [16,17,18,19]. Highly porous scaffolds with nanotopography can mimic the naïve ECM architecture by aiding good interaction and establishing an intercellular connection between the substrate and the cell [20,21,22]. 

Synthetic polymer-based scaffolds are often surface-functionalized with cardiac ECM proteins such as collagen, gelatin, fibronectin to enhance cell proliferation adhesion and differentiation [23,24]. Collagen is the most abundant protein of ECM. The glycosaminoglycans which connects the collagen matrix acts both on structural and non-structural factors for integrity and stability [25,26]. The influence of orientation of polyurethane (PU) and hybrid PU/Collagen random and aligned nanofibers on cell proliferation and adhesion was demonstrated in smooth muscle cells [27]. PLLA scaffold, surface modified with type I collagen was investigated for cell attachment and proliferation [28]. Gelatin, a derivative of collagen formed as byproduct of denaturation of collagen I used to surface functionalize nanofiber substrate has shown to modulate stem cell fate [25,29,30,31,32]. The nanofiber with poly(ε-caprolactone) (PCL) and PCL–gelatin fiber orientation has effects on functional gene expression, adhesion and the proliferation of human smooth muscle cells [33]. Fibronectin is a high molecular weight glycoprotein cell adhesion molecule. Fibronectin helps to bind with receptor protein of cell membrane and integrin to form extracellular matrix [34]. These proteins have shown to increase cell alignment for cardiac repair therapy [35,36]. Furthermore, poly L lysine, which has an essential amino acid with one carboxyl and two amino groups has demonstrated application in tissue engineering grafts. There are exhaustive reports that show the potential use of PLLA scaffolds in bone [37], vascular [38,39] and other tissue engineering applications, however, there are very few reports demonstrating the electrospun PLLA fibrous scaffolds in cardiac tissue engineering [40,41,42,43,44]. Still, no reports exist of the utilization of porous electrospun PLLA fibers as scaffolds for adult human cardiac cells. 

In the present study, porous PLLA electrospun fiber scaffolds were fabricated to mimic the naive ECM of heart and were topologically modified with different proteins such as collagen, gelatin, fibronectin and poly L lysine to test cell adhesion and proliferation of human cardiac fibroblasts. 

## 2. Experimental Section

### 2.1. Materials

All materials were purchased from Sigma (Sigma Aldrich, Tokyo, Japan) (Merck) or Wako chemicals (Wako chemical Ltd., Osaka, Japan), Japan unless otherwise stated. Poly (L-lactide) (PLLA) with molecular weight (Mw) of ~80,000–100,000 was purchased from Polyscience Inc. (Warrington, PA, USA). Porcine Gelatin 0.1% was purchased from Merck (Tokyo, Japan). Collagen calf skin type—I 0.1%, Fibronectin 0.1%, Poly L lysine 0.01% were purchased from Sigma Aldrich (Tokyo, Japan). Fibroblast medium 3, Detach kit, adult human cardiac fibroblast was purchased from Promocell (Heidelberg, Germany). Presto Blue cell viability reagent was purchased from Invitrogen (Thermo Fisher Scientific, Waltham, MA, USA). 2’,7’-Dichlorofluorescin diacetate (DCFH-DA) was purchased from Sigma-Aldrich (Tokyo, Japan). Proteome profiler array (ARY007) and Human Ki-67/MKI67 DuoSet ELISA (DY7617-05) were from R&D systems. Zetasizer 100 μm tracer polystyrene microsphere particles were purchased from Malvern (Malvern, UK). 

### 2.2. Methods

#### 2.2.1. Fabrication of Electrospun Porous PLLA Scaffolds

PLLA fibers were fabricated using an electrospinner (Nanon-O1A MECC Co., Ltd., Fukuda, Japan). The PLLA electrospinning solution with a concentration of 11.5 wt% was prepared dissolving PLLA in chloroform/*N*, *N*-dimethylformamide (DMF) in the ratio of 9:1 under stirring for 18 h at 25 °C and subsequently the solution was kept for 4 h for resting prior to electrospinning. The polymer solution was filled in a 10 mL syringe with a blunt-ended 18-gauge needle. A flow rate of 1 mL·h^−1^ was optimized between the needle tip and collector. Distance between the needle and the ground collector was 15 cm. The stainless collector plate was covered with aluminum foil for easy collection of the electrospun fibers. To obtain a stable polymer jet, an optimized voltage (DC, 15 kV) was applied between the needle and the collector. The electrospinning process was carried out at room temperature. After collecting the electrospun fibers on the aluminum foil, the obtained fiber scaffolds were air dried for 72 h to remove the residual organic solvent. The solution and spinning parameters including viscosity, polymer concentration, solvent and flow rate were optimized before fabricating the fibers. Thus, formed electrospun scaffolds were cut into discs of 7 mm diameter using a punch, for further characterizations. 

#### 2.2.2. Surface Functionalization of Electrospun PLLA Scaffolds

To enhance the surface compatibility of the substrates for cell culture, electrospun PLLA fiber scaffolds were surface functionalization by a simple drop coating physical adsorption method. The electrospun PLLA fiber were cut into desired size and UV sterilized for 1 h. After sterilization, protein solutions such as fibronectin (0.1%), collagen (0.01%), gelatin (0.1%) and poly L lysine (0.01%) were drop coated onto the fibers, respectively. The fiber scaffolds were incubated for 1 h at room temperature to aid better adsorption and functionalization of proteins followed by aspiration of residual protein solution. The surface-modified samples were oven dried (37 °C) before further characterization and use. 

#### 2.2.3. Characterization of Electrospun PLLA Fiber Scaffolds

Optical Microscope (Keyence VH-Z500, Keyence International, Mechelen, Belgium) was used to immediately (1–2 min post fiber formation) observe the fiber integrity for optimizing the electrospinning voltage and feed rate to avoid polymer bead formations. Based on the morphology, the parameters such as voltage and feed rate were adjusted to achieve bead-free and continuous fibers. Morphology and size distribution of the bare and protein functionalized PLLA scaffolds were determined using a scanning electron microscope (SEM, JEOL, JSM-7400, JEOL Ltd., Tokyo, Japan). The electrospun fibers were Pt coated and the micrographs were taken at an accelerating voltage of 5 kV with beam current of 20.0 µA. To determine the average diameter and size distribution of the electrospun PLLA fibers, approximately 100 individual fiber diameters were measured randomly on the fiber mat and a size distribution curve was plotted. Surface topography of the bare and protein modified PLLA scaffolds was analyzed using a confocal laser scanning microscope (CLSM) (LEXT OLS3000, Orangeburg, NY, USA). The physiochemical characterizations were assessed by X-ray photoelectron spectroscopy (XPS) and Thermogravimetric analysis (TGA). XPS was performed using ULVAC-PHI, Inc. (Inc. PHI Quantes instrument, Kanagawa, Japan) PHI Quantes instrument with the binding energy of all spectra being recorded from 0 to 1000 eV with pass energies of 69 eV, 140 eV and 280 eV. Al was used for X-ray generation, for the high resolution and wide scan spectra respectively. The thermal degradation characteristics for bare and protein functionalized PLLA scaffolds was performed using Thermogravimetric analyzer (TGA, DTG-60H, Shimadzu, Japan). The measurements were carried out by heating the sample from room temperature to 600 °C at a rate of 10 °C·min^−1^. Nitrogen was used as purge gas at a flow rate of 10 mL·min^−1^. The weight loss was determined using weight loss curve obtained by comparing the initial and final weights of the fibers. Surface contact angle measurements to determine the hydrophilic properties of both bare and protein functionalized PLLA scaffolds were performed by the half angle sessile drop method using Kyowa Interface Sciences Drop Master DM 301, where 1 μL droplet of deionized distilled water was placed on the surface of each sample and the contact angle ‘θ’ was measured. 4-point random measurements were performed on each sample and the results were analyzed using FAMAS interface measurement and analysis system software ver 3.5.4 (Kyowa Interface Science Co., Ltd., Saitama, Japan). The surface zeta potential for bare and protein functionalized PLLA scaffolds were measured using Malvern Zetasizer Nano ZS (Malvern Panalytical, Malvern, UK). All the measurements were performed using deionized water as the dispersant and polyethylene tracer particles of 1 μm diameter. 

#### 2.2.4. In Vitro Fiber Degradation

In vitro fiber degradation studies were carried out to analyze the organization and geometry of the electrospun fiber scaffolds in phosphate buffer saline (PBS) (pH = 7.4) and cell culture media. Seven mm diameter fiber mats with a thickness of 80 μm were placed in 24 well plates and immersing in 1 mL of PBS and kept in a rocking incubator at 37 °C for 24, 48, 72 h, 15 days and 30 days for PBS and 24, 48, 72 h for media, respectively. 72 h was chosen as maximum time point for culture media for two reasons: (1). Since the cells were culture only till 72 h maximum, for all experimental assays and (2). To prevent contamination issues. Residual PBS and media were aspirated at the end of these periods. After washing respective fibers with deionized water, the samples were oven dried (37 °C) for 12 h. The respective weight was calculated using Equation (1) and morphology was observed under scanning electron microscopy (SEM).

(1) $$\mathrm{Weight}\;\mathrm{loss}\;\%=\frac{w_{o}-w_{t}}{w_{0}}$$

where W_o_ is the starting dry weight and W_t_ is the dry weight after PBS/media incubation. 

#### 2.2.5. Cell Culture

Adult Human cardiac fibroblasts (AHCF) were used to study their growth, adhesion and proliferation on the electrospun PLLA scaffolds. AHCF were cultured at cell density of 5 × 10^6^ cells in a specialized fibroblast growth media at 37 °C in a 5% CO_2_ incubator_._ The cells were sub-cultured every 6–7 days or until confluent. Electrospun PLLA unmodified and protein functionalized samples (2 × 2 cm·sq. mats) were UV sterilized for 1 h prior to cell seeding in a 6-well culture plate. A sterilized metal O ring was placed on the fiber mat to avoid floating of fibers in the culture media. The AHCF were seeded at a cell density of 50,000 cells/PLLA sample, inside the confined space of the metal O ring. After a 1 h resting phase to allow cells to settle on the fibers, desired volume of culture media was added to each well and cultured under aforementioned conditions. Cell viability, post culturing (24, 48 and 72 h) on the respective PLLA scaffolds was performed. The percentage cell viability is estimated by the conversion of resazurin in the dye to fluorescent resorufin by metabolically active cells. After the incubation period, 10% of PrestoBlue dye was added to each sample and incubated for 2 h. Thereafter, fluorescence intensity was measured at 530/590 nm using a multi-detection microplate reader (power scan HT microplate reader Dainippon Sumitomo Pharma, Tokyo, Japan). 

The Reactive oxygen species was estimated using 2,7 dichloroflurorescein diacetate (DCFH-DA) dye. 50,000 cells were seeded on the unmodified and different protein modified PLLA scaffolds. 100 μL of 2,7 dichloroflurorescein diacetate (DCFH-DA) dye at a concentration of 10 mM was added to each well and the plates were incubated for 30 min. Thereafter, the absorbance was measured at 480 nm using multi detection microplate reader. The absorbance of the unmodified and protein modified scaffolds was directly proportional to amount of reactive oxygen species (ROS) generated. 

#### 2.2.6. Cell-PLLA Scaffold Preparation for SEM Observation

AHCF cell adhesion on PLLA unmodified and protein modified scaffolds were evaluated by SEM. The culture conditions were similar to as above. After the incubation, cells were treated in 0.1% glutaraldehyde in culture media for 5 min. Then the remaining media was aspirated and treated with 2.0% glutaraldehyde in phosphate buffer saline (PBS) at room temperature for 30 min. Specimens were dehydrated with 50%, 70%, 90% and 100% ethanol for 5 min, respectively and air dried. Prior to SEM image acquisition the samples were Pt coated. 

#### 2.2.7. Ki67 Proliferation and Proteome Profiling Assay

The cell proliferation was assessed using Ki67 marker kit according to the manufacturer’s instructions. Similarly, the proteome profile was also obtained following the respective instructions of the manufacturer. Cell culture conditions were similar to those mentioned above. 

#### 2.2.8. Statistical Analysis

All experiments were performed as three independent experiments in triplicates. All obtained data were expressed as the mean ± standard deviation (SD). 

## 3. Results

PLLA fiber were fabricated by electrospinning technique, further simple drop casting method was used to surface functionalize the electrospun scaffolds to evaluate their purpose as better niche for enhanced cell adhesion and proliferation. 

### 3.1. Fabrication of Porous Fiber Scaffolds

**Figure 1:**
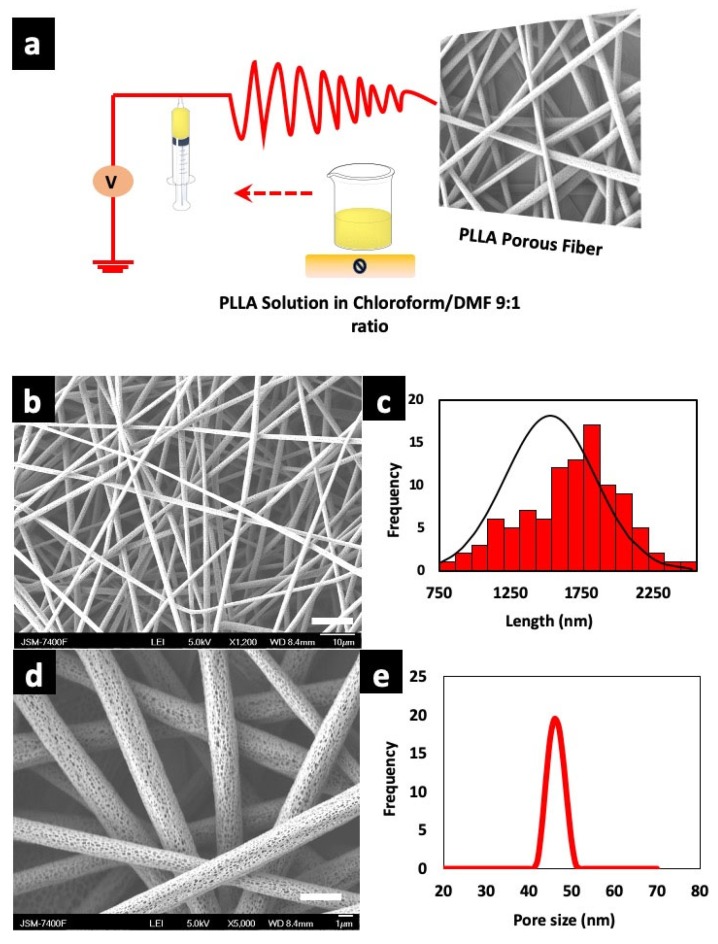
(**a**) Fabrication protocol of poly (l-lactic acid) (PLLA) porous scaffolds and schematic illustration of electrospinning device. (**b**) Scanning electron microscope (SEM) micrographs of as fabricated fibers (scale bar = 10 µm). (**c**) Corresponding diameter distribution of the fibers. (**d**) PLLA fiber with highly porous structure (scale bar = 1 µm). (**e**) Pore size distribution of unmodified fibers as observed under SEM.

Electrospinning is a versatile technique for the fabrication of nano/microfibers. Key parameters such as concentration of the polymer, solution viscosity, temperature, humidity, feed rate, needle gauge, applied voltage, needle to collector distance are taken into consideration for uniform electro spun fibers [45,46]. The right choice of polymer and solvents helps to achieve desired morphology. Herein, we used binary solvent system of chloroform—DMF in a ratio of 9:1 as reported earlier [47]. An ideal solvent for fiber fabrication is one which does not disturb the flow by causing needle blockage due to changes in viscosity. The uninterrupted flow of solution for continuous collection of fiber on the collector was achieved by using binary solvent system. The solvent and processing parameters were optimized for uniform fiber with highly porous structure. We determined an optimum co-solvent ratio of 9:1 for electrospinning solutions of PLLA at 11.5% w/v. A schematic illustration of electrospinning process is shown in Figure 1a. Many previous reports are suggestive that chloroform and DMF are a good solvent system in comparison with other solvents for porous fiber fabrication due to the change in entanglement ratio [48], wherein the high vaporization rate of chloroform is overcome by DMF with a low vapor pressure which helps to lower the drying of electrospinning solution, thus preventing needle blockage [49]. Figure 1b,d show uniform distribution of random fiber architecture with interconnected pores as depicted in the SEM micrograph. The average diameter of the porous PLLA fibers were from 1.58–3.03 µm as shown in Figure 1c. The polymer concentration greatly contributes to the porosity. The pores are formed during evaporation of condensed moisture on the fiber due to the higher molecular weight of PLLA, which limits the solution to fill the pores. Hence, pores are formed where the polymer is absent. The pores are formed on surface of the fibers with increase in humidity, that is, pore size is directly proportional to relative humidity [50]. The SEM micrograph showed an average visible pore size of 40–50 nm on randomly selected individual fibers, which is plotted in Figure 1e. The average thickness of the porous scaffold (unmodified) was ~80.0 ± 0.30 µm as measured with Nikon Digimicrostand. 

### 3.2. Morphology of Surface Functionalization Porous Scaffolds with ECM Mimetic Proteins

**Figure 2:**
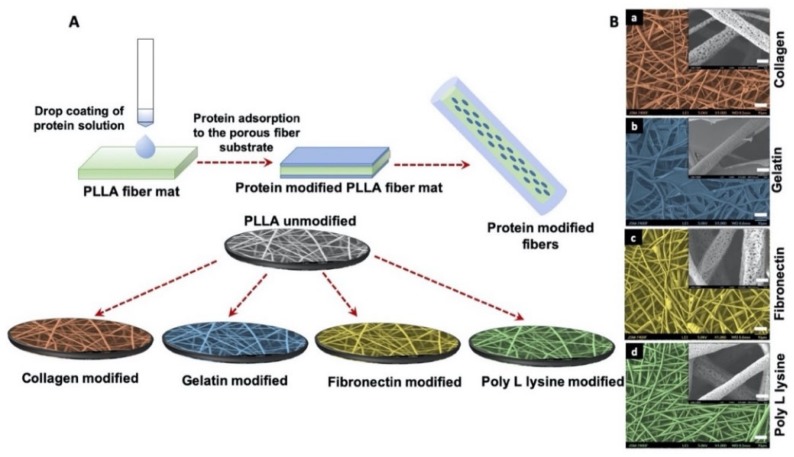
(**A**) Schematic illustration of protein functionalization on porous PLLA scaffolds. Simple drop coating of extracellular matrix (ECM) proteins to porous fiber surface was used for surface functionalization. (**B**) SEM micrographs of protein functionalized porous PLLA scaffolds (false color coded) (scale bar = 10 µm). The inserts are corresponding magnification of 5000× (scale bar = 1 µm). The magnified surface of fiber shows that pores of (**a**) PLLA collagen (**c**) PLLA Fibronectin (**d**) PLLA Poly L lysine are distinctively visible whereas (**b**) PLLA Gelatin fiber scaffold is covered with a layer of gelatin sheet.

Surface-modification of electrospun scaffolds were achieved by drop casting ECM mimetic proteins such as collagen, gelatin, fibronectin and poly l-lysine as shown in Figure 2A. The surface of PLLA collagen fibers was wrinkled and observed to be friable as analyzed by SEM, possibly due to the presence of 0.1 M acetic acid which is used to dissolve collagen (Figure 2B(a)). In comparison with collagen modified fibers, PLLA gelatin fibers formed a gel-like structure with a stable fibrous backbone as shown in Figure 2B(b) whereas, in the case of PLLA fibronectin, residues of protein with webbed morphology were observed on the surface (Figure 2B(c)). However, poly L lysine coated fiber surface retained the original morphology and integrity (Figure 2B(d)) similar to unmodified PLLA fibers when compared with other protein modified counterparts. These observations provide evidence of surface modifications of the PLLA fibers along with providing valuable insights into the related topographical alterations which could in turn have significant effects on the future cellular interactions. 

**Figure 3:**
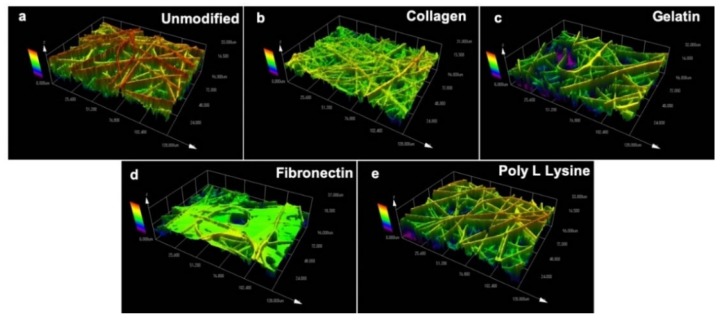
Surface topography of (**a**) unmodified PLLA scaffold and ECM protein surface functionalized PLLA scaffolds (**b**) Collagen, (**c**) Gelatin (**d**) Fibronectin (**e**) Poly L Lysine. The collagen, gelatin and fibronectin functionalized protein scaffold has masked the fiber with a thin film like protein sheet. The topography of collagen, gelatin, fibronectin scaffolds have significantly changed in contrast to the surface topography of poly L lysine and unmodified scaffolds.

Topographical parameters play a pivotal role in directly influencing the cell response and regulating cell growth [51]. Therefore, topography of PLLA unmodified and protein modified fibers were evaluated using a laser scanning microscope (Figure 3). As with SEM observations (Figure 2), it could be seen that the surface topography was significantly changed after protein immobilization on the fiber surface. Figure 3a shows the topography of unmodified electrospun fibers with rigid surface morphology whereas the collagen modified fibers appeared to be thinner (Figure 3b) possibly due to the natural property of collagen itself [52]. The gelatin modified fibers were observed to have a thin sheet of gelatin wrapped over the PLLA fibers as seen in Figure 3c. The fibronectin modified samples presented webbed structures engulfing most of the fiber surface (Figure 3d). However, the poly L lysine modified fibers were in close resemblance with unmodified PLLA fiber scaffold and the surface pores were visible even after the modification. As for the fiber mat thickness post-protein modification, the values were similar to that of the unmodified samples with insignificant variations. 

### 3.3. Physiochemical Characterization

**Figure 4:**
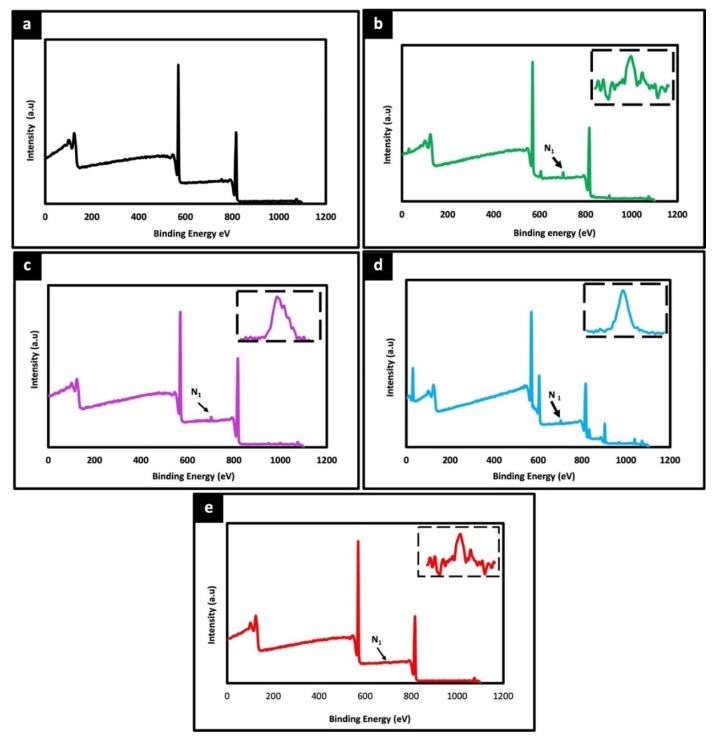
X-ray photoelectron spectroscopy (XPS) wide spectra of unmodified PLLA scaffold (**a**) and different ECM protein surface functionalized PLLA scaffolds (**b**) Collagen, (**c**) Gelatin (**d**) Fibronectin (**e**) Poly L Lysine. The peaks at 285 eV, 532 eV corresponds to C1s and O1s, respectively. A prominent peak at 400 eV for all surface functionalized scaffolds (arrow) represents N1s attributing to successful functionalization of protein on the fiber scaffolds. Inset spectra represent the corresponding nitrogen peak.

The surface chemical composition of electrospun unmodified and protein modified fiber were analyzed by XPS. Figure 4 shows the wide scan spectra of PLLA unmodified and different protein modified samples. Two peaks corresponding to C1s (285 eV) and O1s (532 eV) were observed with all the samples. Additionally, a distinct N1s peak at 400 eV was observed in all protein modified samples (Figure 4b–d), whereas no such peak was detected in unmodified PLLA (Figure 4a). As the unmodified PLLA fibers do not harbor any source of Nitrogen species themselves the N1s signal is bound to be due to the protein modification, thereby confirming the successful immobilization of proteins on the fiber surface, correlating with the microscopic observations in Figure 2 and Figure 3. Further, the thermal stability of unmodified and protein modified PLLA scaffolds were examined by DTA and TGA. The decomposition temperature depicts the maximum processing temperature of each sample. The T onset temperature of sample is calculated as the temperature at which the sample exhibits 1% weight loss. The corresponding thermograms of DTA and TGA are shown in Figure S1a,b. In comparison with protein modified and unmodified fibers, not much significant difference was observed in thermal degradation behavior. However, all the scaffolds exhibited a thermal degradation initiation at slightly different temperatures, unmodified-337.43 °C, collagen-322.70 °C, gelatin-318.67 °C, fibronectin-310.54 °C and poly L lysine-335.98 °C. As the initial weight of all samples were constant, this slight shift in the initiation of thermal degradation can be attributed to the protein immobilized on the surface of the fibers. Also, there is no distinct difference in the thermal behavior of protein functionalized fibers which could be associated with low concentration blend of surface functionalized proteins. 

### 3.4. Surface Wettability and Zeta Potential

**Figure 5:**
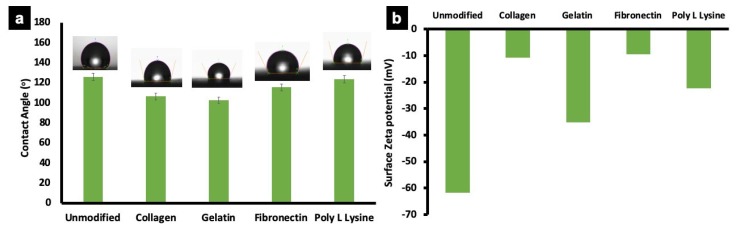
Wettability and zeta potential of unmodified PLLA scaffold and ECM protein surface functionalized PLLA scaffolds. (**a**) Representative average contact angle of unmodified PLLA scaffolds and protein functionalized scaffolds. The water contact angle of unmodified and poly L lysine was relatively similar. However, the collagen, gelatin and fibronectin modified fibers show a slightly reduced wettability behavior, possibly due to the inherent nature of individual proteins. (**b**) The graph represents average zeta potential of unmodified PLLA scaffold and protein functionalized scaffolds. The surface zeta potential of unmodified scaffolds was higher than other samples, with collagen and fibronectin scaffolds showing lowest values.

Wettability (Surface hydrophobicity) of unmodified and protein modified fiber scaffolds were determined by sessile drop method. Water contact angle on a substrate reflects its hydrophilic/hydrophobic nature, which is a crucial factor influencing cell adhesion and protein absorption on any substrate. Surface hydrophobicity greatly influences the cell behavior on a scaffold [53,54]. The water contact angle of unmodified PLLA fiber was 125.5 ± 7.5° depicting its hydrophobic nature (Figure 5a). Similarly, the collagen and gelatin modified fibers showed contact angles of 106.2 ± 2.5° and 102.7 ± 2.5° respectively, which could be attributed to the change in surface characteristics after protein modification as confirmed by zeta potential measurements (Figure 5b), −61.7 (unmodified), −10.7 (PLLA collagen), −35.7 (PLLA gelatin), −9.5 (PLLA fibronectin) and −22.5 (PLLA lysine). However, the water contact angle of poly L lysine and fibronectin coated samples showed no significant decrease (115.5 ± 25° and 123.4 ± 6.5°, respectively). It could be assessed that the unmodified and protein modified substrates are hydrophobic with contact angles ranging from ~122°–102°. Previous reports are suggestive that hydrophobic polymer matrix greatly supports cell attachment in comparison with highly hydrophilic natural and synthetic materials such as cellulose with lower contact angle of ~18° [55]. Additionally, the cumulative results from SEM, confocal laser scanning microscope (CLSM), wettability and zeta potential substantially prove that the proteins are successfully immobilized on the surface of the fibers. 

### 3.5. In Vitro Degradation and Weight Loss

**Figure 6:**
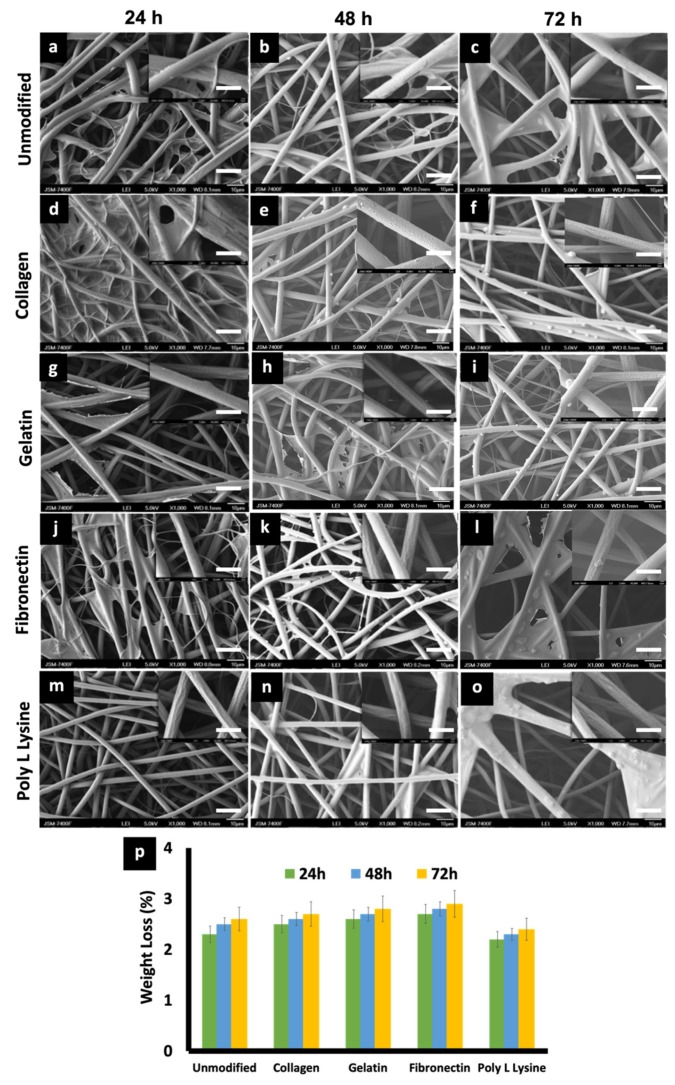
(**a**–**o**) SEM micrographs depicting the morphological features of (**a**) 24 h, (**b**) 48 h, (**c**) 72 h, PLLA unmodified electrospun scaffold, (**d**) 24 h, (**e**) 48 h, (**f**) 72 h PLLA collagen, (**g**) 24 h, (**h**) 48 h, (**i**) 72 h] PLLA gelatin, (**j**) 24 h, (**k**) 48 h, (**l**) 72 h, PLLA fibronectin (**m**) 24 h, (**n**) 48 h, (**o**) 72 h and PLLA poly L lysine scaffolds after being immersed in Cardiac fibroblast media for 24, 48, 72 h at 37 °C. (scale bar = 10 µm). Insets are magnified versions of the corresponding surface (scale bar = 1 µm). serum protein adhesion, in the form of films can be observed in the samples. (**p**) Weight loss percentage of unmodified and protein modified scaffolds after in vitro degradation studies in media for 3 days.

**Figure 7:**
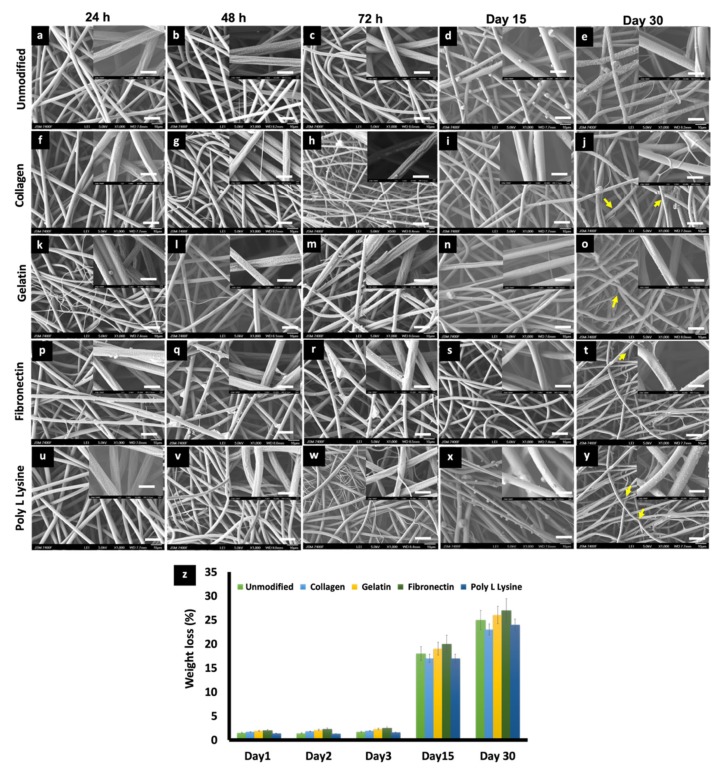
SEM micrographs depicting the changes in morphology of (**a**) 24 h, (**b**) 48 h, (**c**) 72 h, (**d**) Day 15, (**e**) Day 30 PLLA unmodified, (**f**) 24 h, (**g**) 48 h, (**h**) 72 h, (**i**) Day 15, (**j**) Day 30, PLLA collagen, (**k**) 24 h, (**l**) 48 h, (**m**) 72 h, (**n**) Day 15, (**o**) Day 30, PLLA gelatin, (**p**) 24 h, (**q**) 48 h, (**r**) 72 h, (**s**) Day 15, (**t**) Day 30, PLLA fibronectin and (**u**) 24 h, (**v**) 48 h, (**w**) 72 h, (**x**) Day 15, (**y**) Day 30. PLLA poly L lysine electrospun scaffolds after being immersed in PBS 7.4 for different time point at 37 °C at Day 0, Day 2, Day 3, Day 15, Day 30. (scale bar = 10 µm). Inset images are magnifications of the corresponding surface (scale bar = 1 µm). Salt crystal accumulation on the fiber surface can be observed along with the thinning of fibers due to gradual degradation after 30 days of incubation in PBS. (indicated by yellow arrows) (**z**) Weight loss percentage of electrospun PLLA and different protein functionalized scaffolds during 30 days of degradation in phosphate buffer saline (PBS).

The effects of physiological media such as PBS and culture media on the porous PLLA scaffolds were investigated to observe any possible change in the degradation behavior and related morphology of the respective fibers. On primary observation, in the case of media incubation, a film, possibly of the media serum proteins, was found to be adhered to the surface of all the scaffold variants (Figure 6), whereas in the case of PBS, mostly salt (NaCl) crystals were observed to be sticking to the fibers (Figure 7). PLLA fibers degradation was observed over a period of 30 days as a controlled and sustained degradation [56]. The results show a gradual degradation of the fibers (Figure 6 and Figure 7). PLLA unmodified fibers and protein modified fibers did not show any significant change in the porous structure or morphology after 72 h in media or PBS (Figure 6 and Figure 7, Figure S2). However, after 30 days of immersion in PBS slight degradation was observed (Figure 7e,j,o,t,y). Additionally, the % weight loss of the scaffolds was measured at set time points (Figure 6p and Figure 7z). There was insignificant weight loss in the media-incubated samples (Figure 6p). On the other hand, with PBS incubation, in the case of PLLA unmodified fiber sample, the weight loss percentage was 18% and 25% for Day 15 and 30, respectively (Figure 7z). PLLA collagen fibers exhibited a weight loss percentage of 17%, 23% and PLLA gelatin samples with 19%, 26% for 15 and 30 days, respectively. PLLA fibronectin samples had a weight loss percentage of 27% and 25% whereas the poly L lysine samples had a loss percentage of 27% and 24% for day 15 and 30, respectively. Even though insignificant weight loss was observed in culture media for a period of 3 days, on the contrary, nearly 23–25% weight loss was observed after 2 weeks in PBS but with nil to minimum structural deformities (Figure 7). The sustained and controlled degradation properties of PLLA while maintaining structural integrity, as observed in this and previous reports, projects it as a material ideal for tissue engineering applications [56,57].

### 3.6. Cell Adhesion and Proliferation 

**Figure 8:**
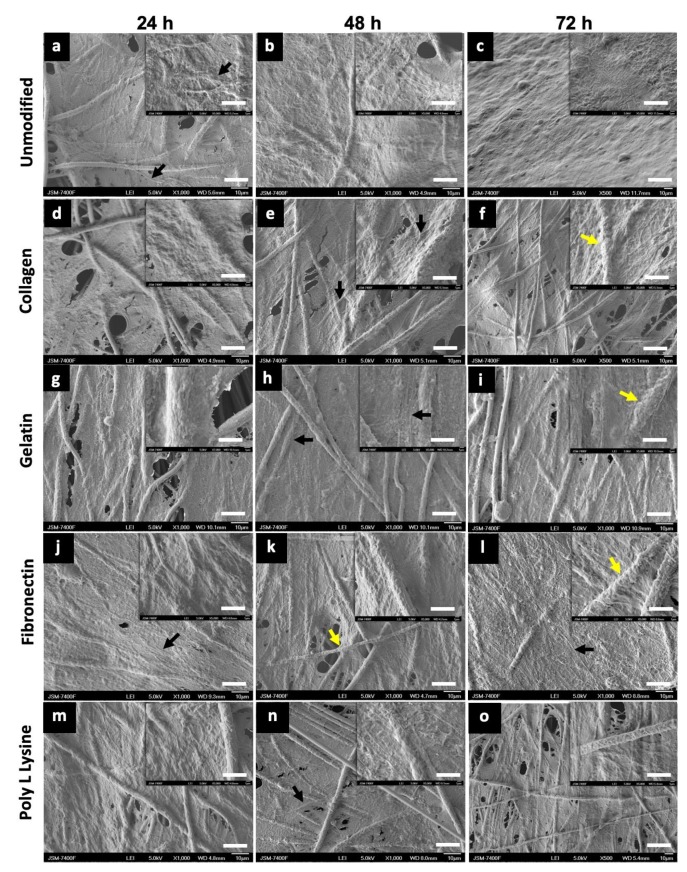
Scanning electron microscope (SEM) images of AHCF cultured on unmodified PLLA and protein modified fibers (scale bar = 10 µm). Adult human cardiac fibroblasts (AHCF) cells can be seen freely spreading on the entire stretch of the scaffolds. The extensive filopodia (black arrow) extended to long distances interconnecting numerous cells and forming a complex network. Fibers can be seen completely engulfed (yellow arrows) by the cellular bodies. These observations suggest the high compatibility of the scaffolds towards the attachment and proliferation of cells. Insets are magnifications of the corresponding surface (scale bar = 1 µm). (**a**) 24 h, (**b**) 48 h, (**c**) 72 h, PLLA unmodified, (**d**) 24 h, (**e**) 48 h, (**f**) 72 h, PLLA collagen, (**g**) 24 h, (**h**) 48 h, (**I**) 72 h, PLLA gelatin, (**j**) 24 h, (**k**) 48 h, (**l**) 72 h, PLLA fibronectin, (**m**) 24 h, (**n**) 48 h, (**o**) 72 h, PLLA Poly L Lysine.

To understand the cell morphology in accordance to the surface topography, cell behavior, cell–material interactions and also, to assess the biocompatibility of the substrate for cell adhesion and proliferation on unmodified PLLA and protein modified PLLA fibers, AHCF cultured on these substrate for 24, 48, 72 h were analyzed by SEM. SEM observations were prioritized over actual cytocompatibility assays to first visually confirm the presence, attachment and growth of the cells on the respective substrates. Irrespective of surface modifications, the AHCF cultured on fiber substrates exhibited a spindle shaped flat morphology, which is unique key feature of the cardiac fibroblasts (Figure 8). Cell attachment to the PLLA substrates was prominently visible after 24 h with excellent cell-biomaterial interaction. The cells spread quickly over the PLLA unmodified and protein modified scaffolds. The cells were able to expand and completely cover the fiber matrix (Figure 8c and Figure S3c) within 72 h of incubation. Cell spreading and proliferation was observed for all the fiber matrix, forming a 3D sheath like architecture with numerous filopodia extending inwards into the fiber scaffold. SEM observations of all the fiber samples revealed cells with well-spread cell body, indicative of the strong cytoskeleton stretching. Cell membrane protrusions with an extensive network of inter-cellular connections were seen. It was interesting to observe that PLLA unmodified scaffolds had an equally good cell adhesion (Figure 8a–c) in comparison with the PLLA protein modified substrates (Figure 8d–o), which could be due to the rough topographic features providing efficient anchorage for the cells. The observed cell behavior can be attributed to the random fibers orientation which enables more interconnecting space for cell proliferation in addition to the porous architecture which provides additional anchorage spots for the cells (Figure S3a,b). The cells were observed to infiltrate into and below the surface of the fiber mat with numerous filopodia probing the pores on the fibers for grip. It has to be mentioned that though the entire surface of the scaffold was covered with cells, the cell did not peel off (till 72 h) indicative of the strong cell-scaffold interaction. A coherent interface between the electrospun fiber and cells is often due to the cellular filopodia protrusions which aids cell migration and elongation on the 3D network of fibers. Such observed cell behaviors on the fibers confirms that the electrospun PLLA scaffolds mimic the naïve extra cellular matrix (ECM) facilitating for effective cell anchorage and sustenance. 

To understand the cell morphology in accordance to the surface topography, cell behavior, cell–material interactions and also, to assess the biocompatibility of the substrate for cell adhesion and proliferation on unmodified PLLA and protein modified PLLA fibers, AHCF cultured on these substrate for 24, 48, 72 h were analyzed by SEM. SEM observations were prioritized over actual cytocompatibility assays to first visually confirm the presence, attachment and growth of the cells on the respective substrates. Irrespective of surface modifications, the AHCF cultured on fiber substrates exhibited a spindle shaped flat morphology, which is unique key feature of the cardiac fibroblasts (Figure 8). Cell attachment to the PLLA substrates was prominently visible after 24 h with excellent cell-biomaterial interaction. The cells spread quickly over the PLLA unmodified and protein modified scaffolds. The cells were able to expand and completely cover the fiber matrix (Figure 8c, S3c) within 72 h of incubation. Cell spreading and proliferation was observed for all the fiber matrix, forming a 3D sheath like architecture with numerous filopodia extending inwards into the fiber scaffold. SEM observations of all the fiber samples revealed cells with well-spread cell body, indicative of the strong cytoskeleton stretching. Cell membrane protrusions with an extensive network of inter-cellular connections were seen. It was interesting to observe that PLLA unmodified scaffolds had an equally good cell adhesion (Figure 8a–c) in comparison with the PLLA protein modified substrates (Figure 8d–o), which could be due to the rough topographic features providing efficient anchorage for the cells. The observed cell behavior can be attributed to the random fibers orientation which enables more interconnecting space for cell proliferation in addition to the porous architecture which provides additional anchorage spots for the cells (Figure S3a,b). The cells were observed to infiltrate into and below the surface of the fiber mat with numerous filopodia probing the pores on the fibers for grip. It has to be mentioned that though the entire surface of the scaffold was covered with cells, the cell did not peel off (till 72 h) indicative of the strong cell-scaffold interaction. A coherent interface between the electrospun fiber and cells is often due to the cellular filopodia protrusions which aids cell migration and elongation on the 3D network of fibers. Such observed cell behaviors on the fibers confirms that the electrospun PLLA scaffolds mimic the naïve extra cellular matrix (ECM) facilitating for effective cell anchorage and sustenance. 

### 3.7. Cytocompatibility of Scaffolds

**Figure 9:**
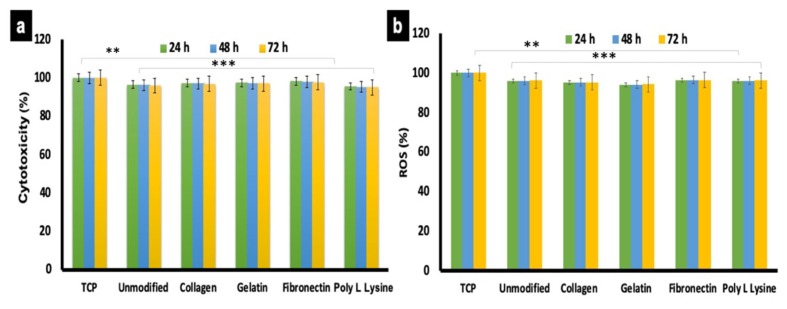
Cytotocompatibility and reactive oxygen species (ROS) generation analysis of AHCF cultured on unmodified and protein functionalized PLLA scaffolds. (**a**) No significant toxicity was observed with any of the scaffold variant. AHCF cultured on TCP were used as positive controls. (**b**) The AHCF cells cultured on the scaffolds did not show any noticeable ROS production, providing evidence that the scaffolds do not exert any stress to cells. (n = 3, ** *p* < 0.01, *** *p* < 0.001).

Once visual confirmation of cellular presence on the scaffolds was obtained (Figure 8), further cytocompatibility tests were conducted. AHCF were cultured on PLLA unmodified and protein modified substrates for 24, 48 and 72 h. Cells grown on conventional tissue culture plastic (TCP) were used as controls. The results were indicative that surface modification did not exert any deleterious effects on the cells (Figure 9a). The cellular viability for protein modified and unmodified sample were above 90% at all time points and on par with the controls. However, a slight (negligible) decrease in cell viability was observed after 48 and 72 h on PLLA scaffolds. Furthermore, the cell adhesion to the fiber substrate arbitrates via strong interactions and chemical composition of the biomaterial matrix and ECM composition [58]. Thus, our results are suggestive that none of the scaffolds exerted any apparent toxicity to the cells and exhibit excellent biocompatibility. These results are correlative of the SEM observations (Figure 8) where nearly all the cells observed did not show any signs of cytoplasmic (cell body) shrinkage or presence of apoptotic bodies, depicting the viable nature of the cells on the respective scaffolds. 

### 3.8. Reactive Oxygen Species Generation 

The accumulation of reactive oxygen species (ROS) within cells and release into the culture media can be qualitatively analyzed by in vitro cell culture model to better understand the mechanism of cell death. During aerobic metabolism, highly reactive molecules of ROS are generated continuously. Excess ROS production can lead to DNA and protein oxidation, cross linking of proteins and eventually cell death. In mammalian cells, free radical species, such as ROS and reactive nitrogen species (RNS) are generated during aerobic metabolism [59]. ROS production is accumulated within the cell or into the extracellular environment based on the cell and its behavior depending on cell-material interaction. Therefore, ROS is one of the major stress markers for cells. To investigate the stress effect of the PLLA unmodified and surface functionalized scaffold by ROS production in AHCF cells, DCFH-DA dye was used, which is a specific fluorescent probe which oxidize a wide range of intracellular ROS. The ROS production observed for unmodified and different protein modified scaffolds did not exhibit any significant difference (Figure 9b) and were even with the controls (normalized to 100%). The results are suggestive that the electrospun porous PLLA fiber substrates are very good candidates and possess good cytocompatibility for AHCF growth, correlating with the previous experimental observations so far. 

### 3.9. Cell Proliferation on Scaffolds

**Figure 10:**
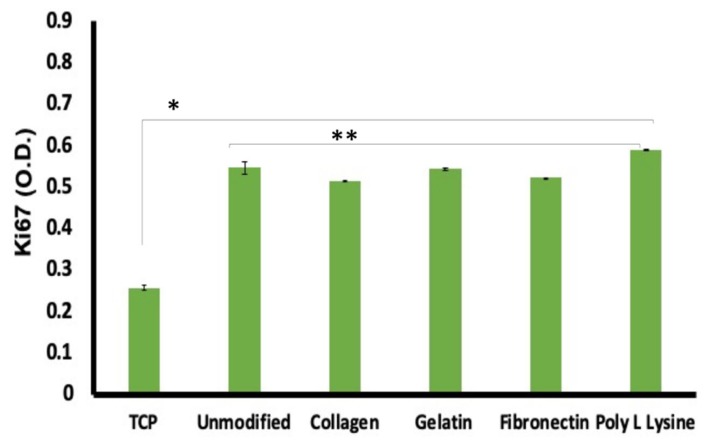
Ki67, a cellular proliferation marker was recorded to be at higher expression levels in cells cultured on the unmodified and protein modified porous PLLA scaffolds than in the tissue culture plastic (TCP) substrates. The expression difference was a significant 2-fold increase in the fiber scaffolds than TCP. These observations are highly supportive of the enhanced compatibility the scaffolds share with the cells than conventional culture ware. (n = 3, * *p* < 0.05,** *p* < 0.01).

We have already established that the AHCF cells are highly compatible with the PLLA scaffolds. Further, to validate the significance of our substrates, we proceeded with a study on the proliferation of cells. Ki67 is a 350–370 kDa nuclear protein linked to mitotic chromosome-associated proteins and is found in all cells except those in the G0 phase of the cell cycle, making it an excellent cell proliferation marker [60]. When analyzed, surprisingly, the PLLA scaffold grown cells exhibited near to twice expression of Ki67 when compared to control group; whereas the Ki67 expression among surface modified and unmodified fibers exhibited insignificant difference (Figure 10). Again, the higher expression of Ki67 in the fiber scaffold groups confirms the ability of the substrate to support increased proliferation kinetics when compared to controls. Though, this observation is in contrast to the equal expression of cell viability (Figure 9a) seen previously, it has to be noted that the cellular dynamics modulated by the surface of cell growth greatly affects the molecular expression profiles, which is witnessed with the Ki67 analysis. Therefore, it can be concluded that though the metabolic profiles of the cells in control and fiber were similar, the molecular expression pattern was significantly enhanced, promoting efficient cell proliferation.

### 3.10. Proteome Profiling

**Figure 11:**
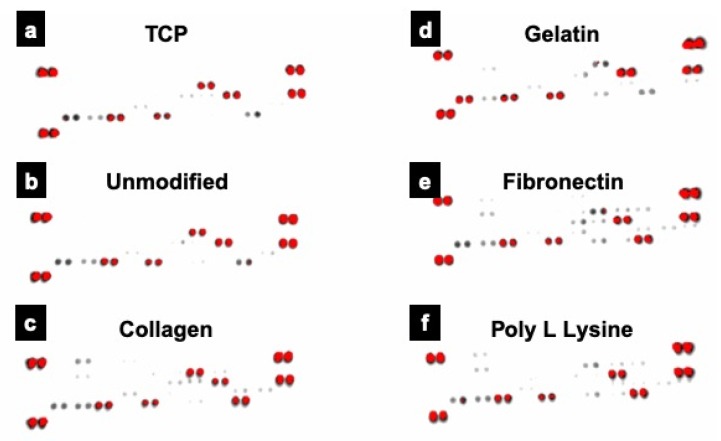
Proteome profile expression of the proteins expressed by cells grown on conventional TCP and unmodified and protein modified PLLA scaffolds. Prominent expression of 8 proteins, crucial for cardiac fibroblast growth and differentiation was observed in various samples analyzed. Of these 4 proteins, IL-1 ß, Pentraxin3, TIMP1 and VEGF were commonly expressed in all the groups. (**a**) Tissue culture plate (TCP), (**b**) PLLA unmodified, (**c**) PLLA collagen, (**d**) PLLA gelatin (**e**) PLLA fibronectin (**f**) PLLA poly L lysine.

With the expression profile for Ki67 varying significantly, we further investigated the metabolic proteome profiling of cardiac fibroblast cells grown on different fiber substratum using array-based proteome profiler. Out of the many analytes (Figure 11), 8 proteins that had major role in cardiac fibroblast growth and differentiation were expressed by different substrates (Table 1 and Table S1). Four analytes, IL-1 ß, Pentraxin3, TIMP1 and VEGF showed expression in all the groups indicating that the nature of cardiac fibroblast and their basic proteome remain unaltered. These proteins were known for their role in myocardial fibroblast post-myocardial infarction, differentiation of mouse embryonic stem cells (mESCs) toward cardiomyocytes, cardiac fibroblast remodeling & migration and myocardial remodeling, respectively. However, cardiac fibroblasts cells grown on collagen coated scaffolds additionally exhibited expression of Activin A and Platelet factor 4 (PF4) proteins. Activins are known for their cell differentiation activity whereas PF4 is known for fibroblast development and is uniquely expressed in the adult heart. On the other hand, fibronectin coated scaffolds grown cells exhibit expression of Activin A, Fibroblast growth factor (FGF-B) and Platelet derived growth factor (PDGF AA). FGF-B is known for promoting myofibroblast conversion and proliferation as well to modulate ECM protein expression. The expression of most of these proteins are positively correlated with discrete role in maintenance/proliferation/differentiation fate of fibroblasts [61] indicating that use of these porous PLLA fiber substrates could help in mimicking in vivo myocardial ECM mesh. 

Also, there are many different analytes expressed by cells grown on different substrates as shown in Table S2. The variant protein profiles of each substrate indicate that the mechanotransduction pathways are variant with each surface modification type. Most of the proteins shown in Table S2 are angiogenic proteins that are involved in endothelial and pericyte proliferation/activation/migration. It indicates that these specific substrates grown fibroblast cells can activate select angiogenic proteins that could act by paracrine action on endothelial/pericytes. This indicates that these electrospun fiber substrates could help in homotypic and heterotypic cell interactions contributing to the organized structure and proper functional analysis [62]. Apart from the exciting prospects of humanized organoids-on-a-chip, in vitro multicellular cardiac co-culture models, in vivo regenerative cardiac patches, cardiac tissue engineering, these scaffolds could also play a crucial role in small to large scale drug discovery processes. 

## 4. Conclusions

In order to identify an ideal cardiac tissue engineering scaffold material, PLLA scaffolds were fabricated by the electrospinning technique with random orientation, highly interconnecting porous architecture exhibiting high specific surface area and porosity to accelerate the exchange of nutrients and oxygen, thereby enhancing the cell attachment and proliferation. Beside the topography and structural parameters, we also investigated the effects of the surface functionalization using ECM mimetic proteins on cell attachment and proliferations. A point to mention is that this is the first report of using porous electrospun PLLA fibers as scaffolds for adult human cardiac cells, thus paving the fundamental pathway for future research on similar lines. The PLLA fibers showed excellent cell attachment and proliferation characteristics as confirmed by various assays. The cells were highly compatible with the scaffolds, irrespective of the surface modifications. The cells were observed to grow extremely well and in a highly organized manner, reminiscent of the in vivo scenario, utilizing the 3D scaffold as an ECM niche. The reactive oxygen species analysis revealed negligible stress to the cells on the scaffolds but were rather highly metabolically active with an enhanced proliferation index. Proteome profiling revealed the expression of certain proteins with crucial role in cardiac fibroblast growth and differentiation in the cells cultured on the scaffolds. To sum up, most of the proteins that are predominantly expressed by cells grown on collagen/fibronectin coated porous PLLA fibers were indeed proteins that have a positive role in extracellular matrix remodeling, fibroblast expansion, differentiation, pericytes proliferation, endothelial cell survival, proliferation and migration, indicating that mechanostimulus induced by the surface topography of substrate can modulate cellular proteome expression rather than just being a mechanical support. Therefore, these scaffolds can be considered effective options for various in vitro and in vivo biomedical applications with an emphasis on the cardiac tissue engineering prospects. 

## Figures and Tables

**Table 1:** Expressed proteins with major role in cardiac fibroblast growth and differentiation.

PROTEINS	TCP	UNMODIFIED	COLLAGEN	GELATIN	FIBRONECTIN	POLY L LYSINE
IL-1 ß	Y	Y	Y	Y	Y	Y
PENTRAXIN3	Y	Y	Y	Y	Y	Y
TIMP1	Y	Y	Y	Y	Y	Y
VEGF	Y	Y	Y	Y	Y	Y
ACTIVIN A	–	–	Y	–	Y	–
PF4	–	–	Y	–	–	–
FGF-B	–	–	–	–	Y	–
PDGF AA	–	–	–	–	Y	–

## Supplementary Materials

The following are available online at https://www.mdpi.com/2073-4360/12/2/451/s1, Figure S1: DTA and TGA curve of unmodified PLLA fiber and ECM protein surface functionalized PLLA fibers; Figure S2: Invitro Degradation: High magnification SEM micrograph of PLLA unmodified fibers and protein modified fibers showing intact pore size after 72 h of incubation in Media and PBS; Figure S3: Scanning electron microscope (SEM) (a,b) and Laser scanning microscope topographic image of (c) AHCF cultured on Unmodified PLLA Electrospun fiber for 72 h, Table S1: Functions of the proteins that have major role in cardiac fibroblast growth and differentiation and were expressed in the scaffolds; Table S2: Angiogenic proteins involved in endothelial and pericyte proliferation/activation/migration that were expressed on the TCP and scaffold grown cells.
