# Supplementary material for: ECM Mimetic Electrospun Porous Poly (L-lactic acid) (PLLA) Scaffolds as Potential Substrates for Cardiac Tissue Engineering
Source: Polymers (Basel). 2020 Feb 14;12(2):451. doi: 10.3390/polym12020451 (PMC7077699; doi:10.3390/polym12020451)
Supplement: Supplementary file 1 [file polymers-12-00451-s001.pdf]

polymers

Supplementary Materials: ECM Mimetic Electrospun Porous Poly (L-lactic acid) (PLLA) Scaffolds as Potential Substrates for Cardiac Tissue Engineering

Priyadharshni Muniyandi [1], Vivekanandan Palaninathan [2], Srivani Veeranarayanan [2], Tomofumi Ukai [1,2], Toru Maekawa [1,2], Tatsuro Hanajiri [1,2] and M. Sheikh Mohamed [1,2,*]

[1] Graduate School of Interdisciplinary New Science, Toyo University, Kawagoe, Saitama, 350-8585, Japan
[2] Bio-Nano Electronics Research Centre, Toyo University, Kawagoe, Saitama, 350-8585, Japan
* Correspondence: sheikh@toyo.jp; Tel.: Phone: +81-492-39-1273/1375, FAX: +81-366-77-1140

Received: date; Accepted: date; Published: date

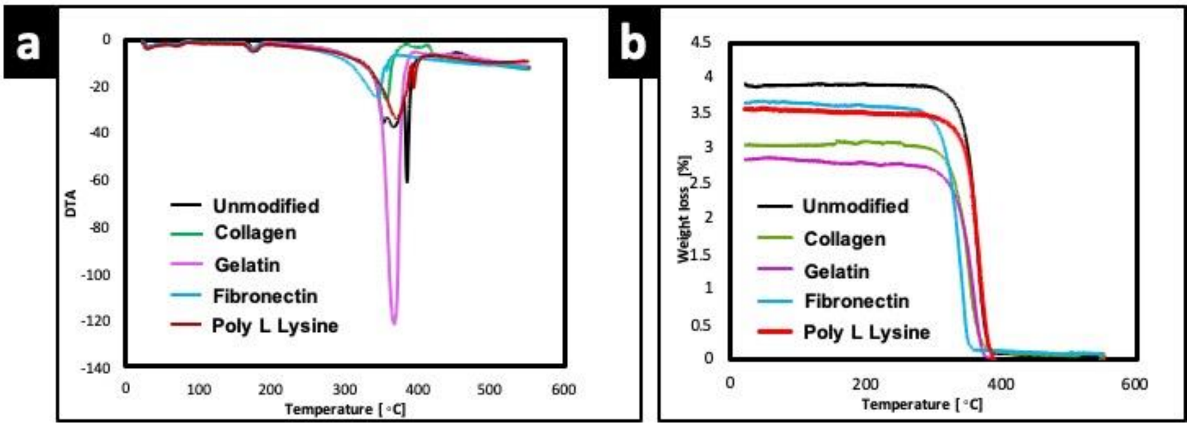

Figure S1. DTA and TGA curve of unmodified PLLA fiber and ECM protein surface functionalized PLLA fibers.

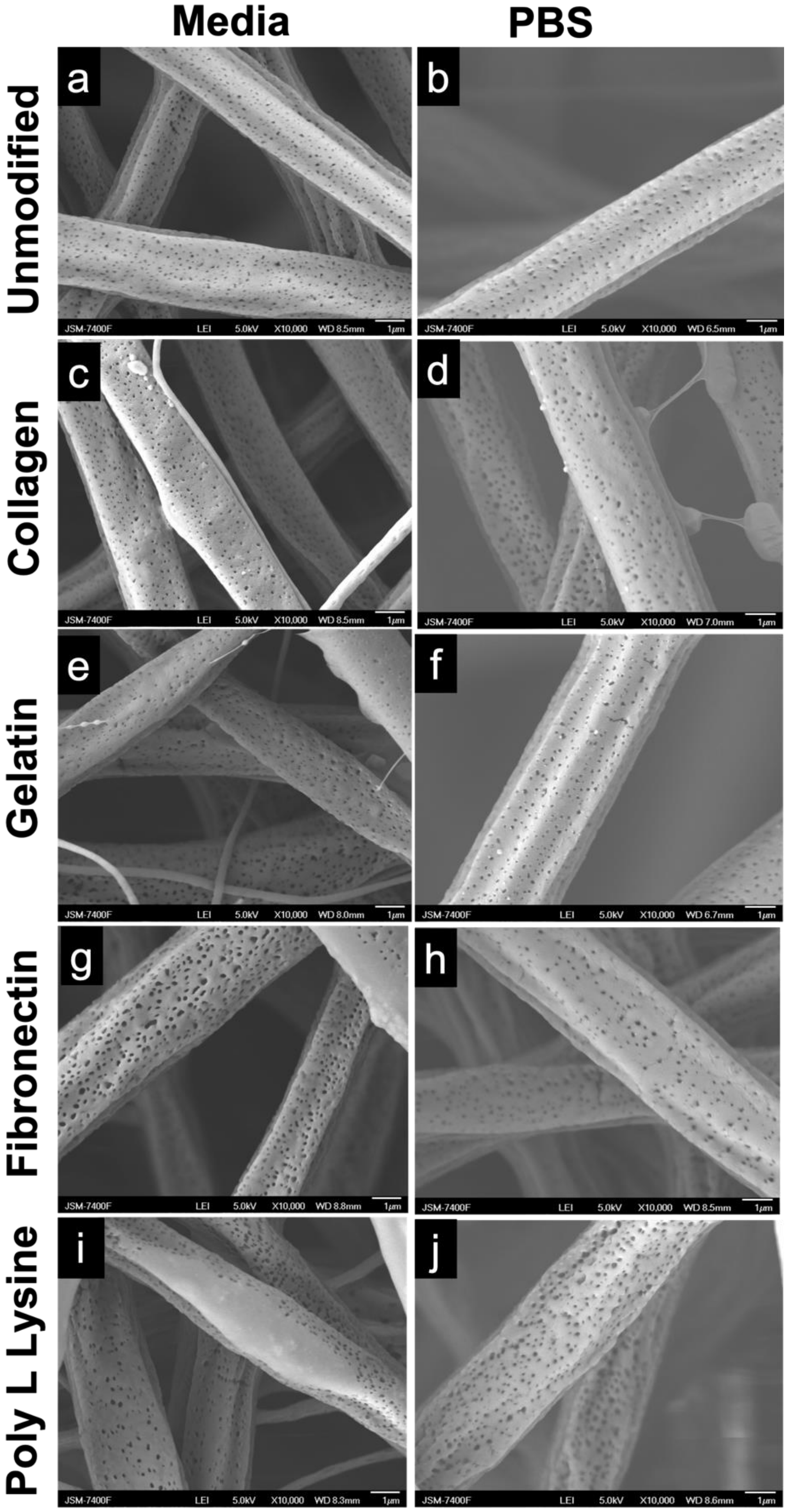

Figure S2. ***In vitro*** **Degradation:** High magnification SEM micrograph of PLLA unmodified fibers and protein modified fibers showing intact pore size after 72 h of incubation in Media and PBS. Scale bar represents 1 µm. (**a**) PLLA unmodified in media (**b**) PLLA unmodified in phosphate buffer saline (PBS), (**c**) PLLA collagen in media (**d**) PLLA collagen in phosphate buffer saline (PBS), (**e**) PLLA gelatin in media (**f**) PLLA gelatin in phosphate buffer saline (PBS), (**g**) PLLA fibronectin in media (**h**) PLLA fibronectin in phosphate buffer saline (PBS), (**i**) PLLA poly l lysine in media (**l**) PLLA poly l lysine in phosphate buffer saline (PBS)

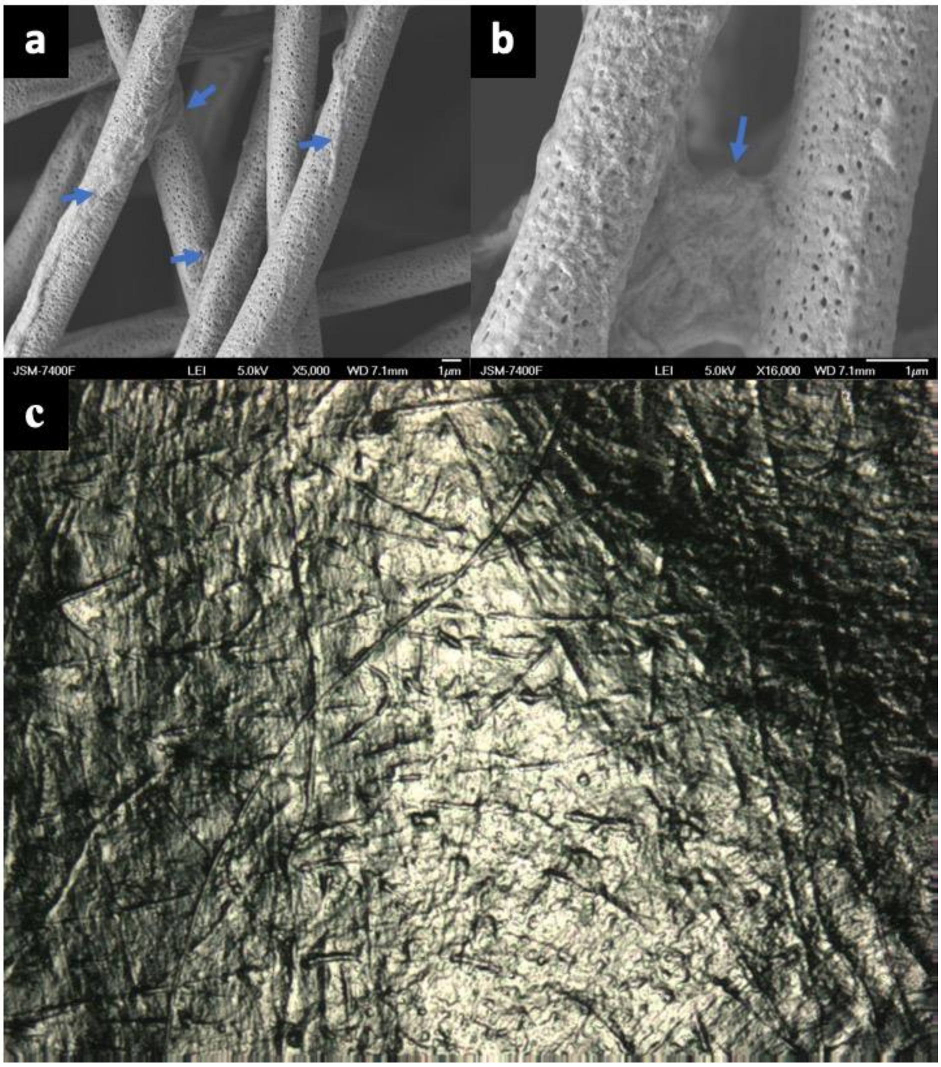

Figure S3. Scanning electron microscope (SEM) (**a**,**b**) and Laser scanning microscope topographic image of (**c**) AHCF cultured on Unmodified PLLA Electrospun fiber for 72 h. In (**a**,**b**) it can be clearly witnessed that the cellular extensions tightly grip and penetrate the fiber structures and also use them as anchors for support (arrows). In the LSM image (**c**) the entire surface of the fiber scaffold is engulfed by cells.

Table S1. Functions of the proteins that have major role in cardiac fibroblast growth and differentiation and were expressed in the scaffolds.

PROTEIN	PROTEIN FUNCTIONS IN CARDIAC FIBROBLAST	OTHER FUNCTIONS
**IL-1 β**	▪ Key inflammatory inducer<br>▪ Promotes cell invasion<br>▪ Repair remodeling of cardiac interstitium<br>▪ Fibroblast proliferation and collagen production<br>▪ ↑ cell migration<br>▪ ↑ ECM degradation<br>▪ ↓ ECM synthesis<br>▪ ↑ Adhesion molecules, proinflammatory cytokines, chemokines<br>▪ ↑ Angiogenesis<br>▪ ↓ Myofibroblast transdifferentiation	▪ Induces prostaglandin synthesis, neutrophil influx and activation<br>▪ Tcell activation and cytokine production<br>▪ B-cell activation and antibody production
**Pentraxin 3**	▪ Promotes cardiac differentiation<br>▪ Biomarker for myocardial infraction	▪ Inflammation, Angiogenesis, tumorigenesis, cell adhesion
**TIMPS**	▪ ECM Homeostasis and remodeling	▪ Inhibitors of Matrix metalloproteinases, a group of peptidases involved in degradation of extracellular matrix<br>▪ Promotes proliferation in wide range of cells types.<br>▪ Anti-apoptotic function.
**VEGF**	▪ Cardiac fibroblasts express both pro-and anti-angiogenic factors that regulated proper regulation of these factors for vascular development and remodeling.	▪ Stimulates the formation of blood vessels<br>▪ Helps in proliferation and migration of vascular endothelial cells<br>▪ Important for physiological and pathological angiogenesis.
**ACTIVIN A**	▪ Promote cell proliferation and collagen synthesis through p38 and ErK MAPK pathways	▪ Regulates morphogenesis stem cell differentiation and organoid formation<br>▪ Wound healing<br>▪ Tubulogenesis of endothelial cell.
**PF4**	▪ Modulates cardiac fibroblast phenotype	▪ Chemotactic for many cell type<br>▪ Inhibitor of hematopoiesis, angiogenesis and T-cell function.<br>▪ Exhibits antimicrobial activity against Plasmodium falciparum.
**FGF B**	▪ ECM remodeling<br>▪ Possess mitogenic and angiogenic activities	▪ Possess mitogenic and angiogenic activities<br>▪ Protein has implications in diverse biological processes such as limb and nervous system development, wound healing and tumor growth

**PDGF AA**	▪ Potent mitogen for fibroblast<br>▪ cell growth, proliferation, and differentiation<br>▪ Regulates the morphogenesis of branching organs such as the prostate, lungs and especially kidney	▪ Helps in proliferation of oligodendrocyte progenitor cells<br>▪ Differentiation of human pluripotent stem cell (hPSC)-derived neural progenitor cells into oligodendrocyte precursor cells

Table S2. Angiogenic proteins involved in endothelial and pericyte proliferation/activation/migration that were expressed on the TCP and scaffold grown cells.

PROTEIN	TCP	UNMODIFIED	COLLAGEN	GELATIN	FIBRONECTIN	LYSINE
ENDOTHELIN	Y	Y	Y	Y	Y	Y
IL-8	Y	Y	Y	Y	Y	Y
MCP-1	Y	Y	Y	Y	Y	Y
IGFBP-1	Y		Y	Y	Y	
SERPIN E1	Y	Y	Y	Y		Y
SERPIN F1	Y	Y	Y	Y		Y
THROMBOSPONDIN 1	Y	Y	Y	Y		Y
DPPIV			Y	Y	Y	
PIGF		Y	Y			Y
FGF-1			Y		Y	
PIGF					Y	
PROLACTIN				Y	Y	
UPA					Y	
VASOHIBIN					Y	
